# Risk of malignancy of cytologic categories and post‐biopsy clinical management of renal oncocytic neoplasms

**DOI:** 10.1002/cncy.70064

**Published:** 2025-12-16

**Authors:** Xiaoqi Lin

**Affiliations:** ^1^ Department of Pathology Northwestern University‐Northwestern Feinberg School of Medicine Chicago Illinois USA

**Keywords:** clinical management cytology, renal oncocytic neoplasm, renal mass biopsy, risk of malignancy

## Abstract

**Background:**

Renal mass biopsy (RMB) cytology is increasingly used for the pretreatment evaluation of renal masses. Cytopathologic classification of RMB specimens facilitates communication with clinicians and provides valuable risk stratification to guide management decisions. This study evaluated the risk of malignancy (ROM) associated with RMB cytology in renal oncocytic neoplasms (ONs).

**Design:**

The renal neoplasms with oncocytic features were reclassified per the 5th edition of the WHO Classification of Tumors. RMB cases were assigned to six diagnostic categories: 1) nondiagnostic (ND), 2) benign, 3) low risk oncocytic neoplasm (LRON), 4) atypical, 5) suspicious for malignancy (SFM), and 6) malignant.

**Results:**

In total, 198 RMB cases with oncocytic features were identified, comprising oncocytoma (111 [56%]), chromophobe renal cell carcinoma (RCC) (55 [28%]), LRON (10 [5%]), hybrid oncocytic neoplasm (8 [4%]), low‐grade oncocytic tumor (7 [4%]), papillary RCC (6 [3%]), and eosinophilic vacuolated tumor (1 [1%]). The overall ROM for ONs was 35%. ROMs by category were ND (29%), benign (2%), LRON (30%), atypical (100%), SFM (50%), and malignant (100%). Post‐RMB resection was more frequently used for malignant (36 of 57 [63%]), SFM (1 of 2 [50%]), and ND (4 of 7 [57%]), whereas active surveillance was more frequently employed for benign ON (100 of 111 [90%]) and LRON (12 of 20 [60%]). Cryoablation was used in 9% (18 of 198) of ONs.

**Conclusions:**

This study defines ROMs across cytologic categories of ONs to stratify the risks and underscores the valuable role of RMB. These findings provide a framework for clinicians to make informed decisions based on cytologic classification and associated ROM.

## Abstract 2

This study demonstrated the risk of malignancy of cytologic categories for renal oncocytic neoplasms and studied the post‐biopsy managements.

## INTRODUCTION

A few types of renal neoplasms may exhibit abundant granular cytoplasm, encompassing benign, low malignant potential, and malignant neoplasms. Renal mass biopsy (RMB) cytology is increasingly used for the pretreatment diagnosis of renal masses. Management guidelines outlined by major organizations, including European Association of Urology (2022), European Society for Medical Oncology (2019), American Urological Association (2021), French AFU Cancer Committee (2022), American Society of Clinical Oncology (2017), and National Comprehensive Cancer Network (2022), recommend management strategies for renal masses including RMB, active surveillance, ablation, surgical resection, and systemic therapies (e.g., chemoradiation, targeted therapy, and immunotherapy).^1^, ^2^, ^3^, ^4^, ^5^, ^6^ Cytopathologic classification of RMB specimens facilitates communication with clinicians, guides clinical management decisions, and significantly contributes to the reduction of overtreatment.^7^, ^8^

Radical or partial nephrectomy has traditionally been the standard treatment for localized renal masses. However, in recent years, increasing numbers of patients have been managed by less invasive approaches such as ablation, active surveillance, chemoradiation therapy, and molecular‐targeted therapies following radiologic imaging studies and RMB in accordance with clinical guidelines.

Establishing a definitive diagnosis of renal oncocytic neoplasms (ONs) is often challenging, due to their overlapping morphologic features, immunohistochemical profiles, and genomic alterations. Data on the risk of malignancy (ROM) and post‐RMB clinical management for ONs remain extremely limited. This study aims to assess the ROMs of cytologic categories and post‐RMB clinical management strategies for ONs in a tertiary care setting.

## MATERIALS AND METHODS

### Case selection

This study was approved by the institutional review board of Northwestern University. In total, 198 RMB cases with diagnoses of ONs were retrieved from the Northwestern Memorial Hospital cytopathology database between July 2002 and April 2024. Diagnoses included oncocytoma, chromophobe renal cell carcinoma (ChRCC), hybrid oncocytic neoplasm (HON), low‐grade oncocytic tumor (LGOT), eosinophilic vacuolated tumor (EVT), papillary renal cell carcinoma (PRCC) with oncocytic features, and oncocytic neoplasm.

The following variables were recorded: sex, age, cytology diagnosis, follow‐up surgical pathology diagnosis, mass size, previous malignant history, clinical management, and metastatic status.

### Fine‐needle aspiration biopsy and core needle biopsy

RMB, including percutaneous fine‐needle aspiration biopsy (FNAB) and/or core needle biopsy (CNB), were performed under guidance of ultrasound or computed tomography (CT) scan using 22‐ or 20‐gauge needles for FNAB and 20‐ or 18‐gauge core needle devices for CNB. A set of FNAB smears and CNB touch preparations were air‐dried and stained with modified Giemsa (Diff‐Quik) stain for rapid on‐site evaluation (ROSE) to access specimen adequacy and render immediate preliminary interpretation by board‐certified cytopathologists. Another set of alcohol‐ or methanol‐fixed FNA smears were stained with Papanicolaou stain for further cytologic evaluation.

### Histology and immunohistochemistry

Pellets from FNAB rinses and aliquots, CNB tissue, and total or partial nephrectomy specimens were fixed in formalin solution, embedded with paraffin, sectioned, and stained with hematoxylin and eosin (H & E) for histologic examination.

Immunohistochemical (IHC) staining was performed on the sections from cell blocks, cores, or resected tissues with appropriate positive and negative controls (i.e., positive controls demonstrated expected antigen expression and negative controls showed no staining). Antibodies used in the study included those against AMACR (p504s, M3616, DakoCytomation), CA9 (SC‐25599, Santa Cruz Biotechnology), CD117 (A4502, DakoCytomation), and cytokeratin 7 (M7018, DakoCytomation), etc. IHC results were graded in a semiquantitative manner and scored as either diffuse (>50% of tumor cells positive), focal (between 5% and 50%), or negative (<5%). Immunoprofiles were used for subclassification of renal neoplasms.

### Cytologic diagnoses, categorization, and calculation of risk of malignancy

Cytologic diagnoses were rendered based on a combination of cytologic and histologic features, immunoprofiles, and available molecular results obtained from cytologic specimens. Renal neoplasms with oncocytic cytoplasm were reclassified according to the 2022 WHO Classification of Tumors (5th edition).^9^ Diagnoses established from nephrectomy specimens were regarded as final diagnoses. In cases without nephrectomy, clinical follow‐up was used to determine the accuracy of the RMB diagnosis. For example, detection of metastasis during active surveillance was indicative of ChRCC, whereas a diagnosis of oncocytoma based on RMB was managed clinically with active surveillance without complications, reflecting a benign clinical course. The correct RMB diagnosis rate is defined as number of correct diagnoses divided by total number and then times 100%.

Based on cytologic findings, all RMB cases were categorized into six diagnostic groups: 1) nondiagnostic/insufficient/inadequate (ND); 2) benign; 3) low‐risk oncocytic neoplasm (LRON) that was originally diagnosed as oncocytic neoplasm (oncocytoma vs. low‐grade ChRCC vs. HON); 4) atypical; 5) suspicious for malignancy (SFM); and 6) malignant. The diagnostic category of each neoplasm was assigned according to the final diagnosis using the latest WHO Tumor Classification, as described above. ND was defined as insufficient material for definitive diagnosis, such as only normal renal components that did not represent the targeted lesion (six cases in this study), rare cells (one case in this study), necrosis, degenerated cells, hemorrhage, etc. Benign ONs include oncocytoma, LGOT, and EVT in this study. LRON was defined as the cytomorphology and/or immunoprofile was not specific for any known specific ON and the differential diagnosis included benign ON versus low‐grade malignant ON. Atypical was defined as presence of scant atypical cells (one case in this study), poorly preserved atypical cells, or poorly stained atypical cells that were insufficient for definitive diagnosis. SFM was defined as presence of atypical cells that were quantitatively (one case in this study) or qualitatively (one case in this study) not enough for malignancy (two cases in this study). Malignant include primary malignant neoplasms.

The ROM of each cytologic diagnostic category was calculated by dividing the number of malignant neoplasms by the total number of cases in that category and then multiplying the result by 100%.

### Post‐RMB clinical management of patients with renal oncocytic neoplasms

Post‐RMB clinical management of patients was recorded and classified into six categories: 1) total or partial nephrectomy, 2) chemoradiation, 3) ablation, 4) active surveillance, 5) not available (NA), and 6) deceased.

### Statistical analysis

Student *t*‐test, *χ* ^2^ test, and Fisher correct test were used for statistical analysis of the data, and results are reported in this article.

## RESULTS

### The biopsied renal oncocytic neoplasms

**FIGURE 1:**
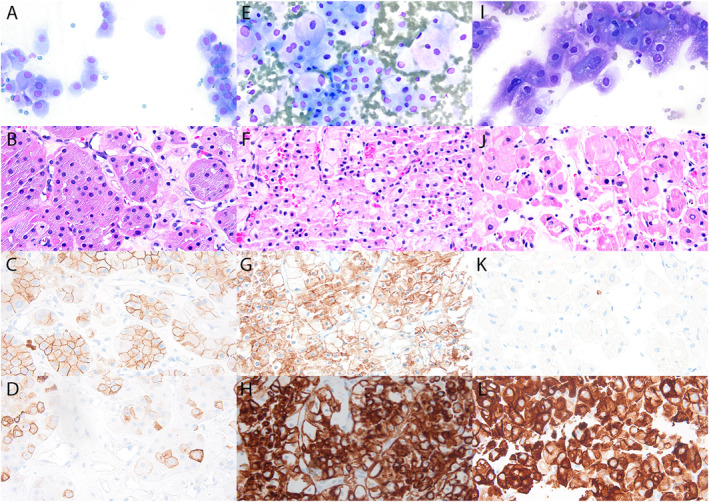
Cytomorphology, histomorphology, and immunostain for CD117 (C, G, and K), immunostain for CK7 (D, H, and L) of oncocytoma (A–D), chromophobe renal cell carcinoma (E–H), and low‐grade oncocytic tumor (I–L), 400×. (A, E, and I) Cytomorphology: smears or touch preparations were stained with modified Giemsa (Diff‐Quik) stain. (B, F, and J) Histomorphology: tissue sections were stained with H & E. Immunohistochemistry: immunostains for CD117 (C, G, and K) and CK7 (D, H, and L) were performed on tissue sections.

In total, 198 RMB cases of ONs were identified (Table 1), which included oncocytoma (111 [56%]) (Figure 1A–D), ChRCC (55 [28%]) (Figure 1E–H), LRON (10 [5%]), HON (8 [4%]), LGOT (7 [4%]) (Figure 1I–L), PRCC (6 [3%]), and EVT (1 [1%]). Two cases were biopsied with FNAB only, 14 cases with combined FNAB and CNB, and 182 cases with CNB with touch preparation only.

**TABLE 1:** The biopsied renal oncocytic neoplasms.

Neoplasm	No. (%)	Accurate Dx, No. (%)	Age, years (Mean ± SD)	Male/female	Size (cm)	Prior neoplasm, No. (%)	Metastasis, No. (%)
Oncocytoma	111 (56)^a^	101 (91)	67.8 ± 10.3	83/28^a^	3.0 ± 1.9	17 (15)	
ChRCC	55 (28)	45 (82)	59.7 ± 12.2	27/28	4.4 ± 3.5	7 (13)	4 (7)
LRON	10 (5)	10 (100)	71.4 ± 11.1	6/4	2.8 ± 0.8	2 (20)	
HON	8 (4)	3 (38)^a^	53.6 ± 18.1	4/4	3.1 ± 1.2	1 (13)	
LGOT	7 (4)	7 (100)	73.7 ± 3.8	3/4	2.9 ± 1.7	1 (14)	
PRCC	6 (3)	6 (100)	75.3 ± 8.2	3/3	5.1 ± 2.8	1 (17)	
EVT	1 (1)	1 (100)	64	0/1	3.7	0 (0)	
Total	198	173 (87)		126/72		29 (15)	

Abbreviations: Accurate Dx, accurate diagnosis rate; ChRCC, chromophobe renal cell carcinoma; EVT, eosinophilic vacuolated tumor; HON, hybrid oncocytic neoplasm; LGOT, low‐grade oncocytic tumor; LRON, low risk oncocytic neoplasm; PRCC, papillary renal cell carcinoma; SD, standard diviation.

^a^ Chi‐square test: *p* < .01.

The accuracy of RMB cytologic diagnosis was as follows: 91% (101 of 111) for oncocytoma, 82% (45 of 55) for ChRCC, 38% (3 of 8) for HON, 100% (7 of 7) for LGOT, 100% (1 of 1) for EVT, and 100% (6 of 6) for PRCC. The overall accuracy of RMB diagnosis for ONs was 87% (173 of 198).

There were no statistical differences in patient age among the different ON subtypes (Table 1). ONs were male predominant (M/F = 1.8/1), largely attributive to the high proportion of renal oncocytomas that were more common in men (M/F = 3/1). Other ON subtypes exhibited no significant sex predilection (Table 1). There were no statistical differences in tumor sizes among ON subtypes, although malignant ONs tended to be slightly larger than benign ONs. Metastasis was observed exclusively in ChRCC (4 of 55 [7%]) during following up, two to the liver, one to the lymph node, and one to the lung. No other ON subtypes showed evidence of metastasis.

A history of prior neoplasms was identified in 13%–20% of both malignant and benign ONs, without statistically significant differences among ON subtypes (Table 1). Prior histories of neoplasms include a wide range of neoplasms, such as breast carcinoma, esophageal carcinoma, hepatocellular carcinoma, lung adenocarcinoma, lymphoma (Hodgkin’s lymphoma, multiple myeloma), meningioma, prostatic carcinoma, Merkel cell carcinoma, renal cell carcinoma, renal oncocytoma, skin squamous cell carcinoma and basal cell carcinoma, small cell carcinoma, and thyroid oncocytic neoplasm.

### The risk of malignancy of cytologic categories for renal oncocytic neoplasms

The most frequent diagnosis among ONs was the benign category (111 of 198 [56%]) (Table 2), including renal oncocytoma, LGOT, and EVT. The malignant category was the second most common (57 of 198 [29%]), including ChRCC, PRCC, and HON. The LRON accounted for 10% of cases. The remaining cytologic categories were less frequent, 4% (7 of 198) for ND, 1% (1 of 198) for atypical, and 1% (2 of 198) for SFM.

**TABLE 2:** The risk of malignancy of cytologic categories for renal oncocytic neoplasms.

Category	No. (%)	ROM (%)
Nondiagnostic	7 (4)	2 (29)
Benign renal oncocytic neoplasm	111 (56)	2 (2)
Low risk oncocytic neoplasm	20 (10)	6 (30)
Atypical	1 (1)	1 (100)*
Suspicious for malignancy	2 (1)	1 (50)*
Malignant	57 (29)	57 (100)*
Total	198	69 (35)

*Note:* *χ* ^2^ test and Fisher exact test.

Abbreviation: ROM, risk of malignancy.

^*^ *p* < .05.

The overall ROM for ONs was 35% (69 of 198) (Table 2), showing a progressive increase across diagnostic categories from 2% (2 of 111) in the benign group to 100% (57 of 57) in the malignant group. The ROMs for LRONs and ND were 30% (6 of 20) and 29% (2 of 7), respectively. Among 103 cases diagnosed as oncocytoma on RMB, two were confirmed as ChRCC on subsequent nephrectomy, yielding a ROM of 2% (2 of 103) for oncocytoma. The diagnostic accuracy for oncocytoma specifically was 98% (101 of 103).

When SFM and malignant categories were grouped as “positive” and ND, benign, LRON, and atypical were grouped as “negative,” the sensitivity, specificity, positive predictive value (PPV), and negative predictive value (NPV) of RMB for ONs was 84.1%, 99.2%, 98.3%, and 92.1%, respectively.

### The post‐RMB clinical management of patients with renal oncocytic neoplasm

Active surveillance was the most common post‐RMB clinical management for ONs (123 of 198 [62% of cases]), followed by nephrectomy (53 of 198 [27%]) and cryoablation (18 of 198 [9%]) (Table 3). Three patients died of non‐neoplastic causes.

**TABLE 3:** The clinical management of each diagnostic category in renal mass biopsy cytology.

Category	No.	Nephrectomy, No. (%)	Ablation, No. (%)	Surveillance, No. (%)	N/A, No. (%)	Deceased, No. (%)
Nondiagnostic	7	4 (57)*	1 (14)	2 (29)	0 (0)	0 (0)
Benign ON	111	7 (6)	2 (2)	100 (90)*	0 (0)	2 (2)
LRON	20	5 (25)	2 (10)	12 (60)*	0 (0)	1 (5)
Atypical	1	0 (0)	0 (0)	1 (100)	0 (0)	0 (0)
Suspicious	2	1 (50)*	0 (0)	1 (50)	0 (0)	0 (0)
Malignant	57	36 (63)*	13 (23)*	7 (12)	1 (2)	0 (0)
Total	198	53 (27)	18 (9)	123 (62)	1 (1)	3 (2)

*Note:* *χ* ^2^ test and Fisher exact test.

Abbreviations: LRON, low‐risk oncocytic neoplasm; N/A, not available; ON, oncocytic neoplasm.

^*^ *p* < .05.

Radical or partial nephrectomy was more frequently performed in patients diagnosed in malignant ONs (36 of 57 [63%]) and those in the ND (4 of 7 [57%]) based on clinical and imaging findings. In contrast, active surveillance was the preferred approach for patients with benign ONs (100 of 111 [90%]) and those categorized as LRON (12 of 20 [60%]). Notably, two of seven (29%) patients with ND diagnosis were also managed with active surveillance. Ablation was primarily used in cases of small, low‐grade malignant ONs confined to the kidney, as well as in selected benign ONs. No patients received chemoradiation alone as the primary post‐RMB treatment modality.

## DISCUSSION

Although it remains debated if RMB is implemented as a standard clinical practice for pretreatment diagnosis, this study demonstrates that RMB effectively stratifies renal oncocytic neoplasms based on the ROMs associated with specific cytologic diagnostic categories. The stratification provides essential guidance for clinical management of ONs, including active surveillance, minimally invasive ablation, or surgical resection.

In this cohort, the overall ROM for ONs was 35% (69 of 198), comparable to a previous study reporting 31.3%,^10^ confirming that most ONs are benign. ROMs varied significantly by cytologic category: 2% (2 of 111) in benign, 29% (2/7) in ND, 30% (6/20) in LRON, 50% (1 of 2) in SFM, and 100% in both atypical (1 of 1) and malignant (57 of 57) groups. In addition, oncocytoma, which constituted most benign ONs, had a ROM of 2% (2 of 103), the same as ROM for benign ONs, reinforcing the accuracy of RMB in identifying benign ONs. Two ChRCCs were misinterpreted as oncocytomas on RMB, indicating the overlap in cytomorphology and immunoprofiles, as details in one of our previous studies.^11^ The high ROMs for atypical, SFM, and malignant categories indicate the accuracy of RMB in identifying malignant ONs. These data demonstrate that RMB can effectively stratify the ONs based on the ROMs of cytologic diagnostic categories.

Because 64% (126 of 198) of ONs were found to have benign clinical behavior, ONs were mainly managed with conservative treatment strategies, active surveillance (123 of 198 [62%]) and cryoablation (18 of 198 [9%]), and less frequently with nephrectomy (53 of 198 [27%]). Active surveillance rate in our medical center is slightly higher than previous studies (46%–54.6%),^12^, ^13^ but is similar to one study (66%).^14^ Active surveillance was more commonly selected for ONs in benign (100 of 111 [90%]) and LRON category (12 of 20 [60%]), and much less likely used for ONs in ND (2 of 7 [29%]) and malignant category (7 of 57 [12%]). Nephrectomy rate (53 of 198 [27%]) is much less frequently used than a previous study (45.4%),^13^ but higher than other studies (7%–19%).^12^, ^15^ Nephrectomy was primarily used in malignant (36 of 57 [63%]) and ND cases (4 of 7 [57%]) but less frequently used in benign ONs (7 of 111 [6%]) and LRON (5 of 20 [25%]). Ablation rate (18 of 198 [9%]) is lower than previous studies (23%–27%).^12^, ^14^ Ablation was reserved for small, low‐grade malignant ONs or benign ONs confined to the kidney. There was no statistical significance in ablation rate among cytologic diagnostic categories. No patients were treated solely with chemotherapy or radiation therapy, consistent with the low metastatic rare (4 of 55 [7%]), observed only in ChRCCs, mostly to local lymph nodes. Seven (12%) malignant cases were managed with active surveillance due to other severe comorbidities or advanced age precluding nephrectomy. The metastatic rate aligned with previously reported rates of 5%–6%.^16^ This study and previous publications demonstrated that active surveillance for biopsy‐proven renal oncocytomas was ontologically safe and patient adherence was high.^14^, ^17^ The oncocytic PRCC has a similar biologic behavior to PRCC type 1^18^ and should be treated as low grade RCC. Data show that clinical management patterns reflect ROMs.

This study demonstrated that ONs can be diagnosed with RMB with high accurate rates ranging from 82% (45 of 55 for ChRCC) to 100% (7 of 7 for LGOT and 6 of 6 for PRCC) except for HONs (3 of 8, 38%) (Table 1). The overall accurate rate for diagnosing ONs was 87% (173 of 198). The accurate diagnostic rate for oncocytoma was 91%, which is close to the highest rate among the previous studies (68%–94%).^12^, ^13^, ^14^ This is probably because the cytologic diagnoses were rendered in our institute by combination of cytomorphology, histology of cell blocks or cores, and immunostains for CD117, CK7, etc.^11^, ^19^ There are still problems with calculation of accurate diagnostic rate, as 18 cases (9%) in this study were ablated and the diagnoses from RMB were considered as final. For these patients, the active surveillance and tumor bed biopsy might appear tumor‐free and therefore might not definitively “declare” the neoplasm as “benign or malignant.” When combining SFM and malignant category as “positive” and ND, benign, LRON, and atypical as “negative,” the sensitivity, specificity, PPV, and NPV of RMB for ONs was 84.1%, 99.2%, 98.3%, and 92.1%, respectively. The high sensitivity, specificity, PPV, NPV, and accurate rate for ONs demonstrated that RMB has implications for the ultimate management strategies the patients will undergo.

This study revealed notable ON subtype distribution: oncocytoma (111 of 198 [56%]) was the most common, followed by ChRCC (55 of 198 [28%]), LRON (10 of 198 [5%]), HON (8 of 198 [4%]), LGOT (7 of 198 [4%]), PRCC (6 of 198 [3%]), and EVT (1 of 198 [1%]). Compared to prior studies, the frequency of oncocytoma (56%) was slightly lower than previous studies (64.6%–67%),^10^, ^19^ whereas ChRCC (28%) was higher than previous studies (12%).^10^, ^19^ HON accounted for 4% in this study, which was slightly less than a previous study.^10^ The combination of malignant oncocytic neoplasm (ChRCC and HON) accounted for 32%, lower than another study (50%).^14^ LGOT’s prevalence (4%) aligned with a prior study (4.2%) of oncocytoma and 0.35% of nephrectomy renal neoplasms.^20^ These findings further support RMB’s role in identifying diverse ON subtypes when integrating cytomorphologic, histomorphologic, immunohistochemical, and possibly molecular data.

The differential diagnosis for ONs also includes those benign or malignant renal neoplasms with granular cytoplasm, such as high‐grade RCC (HG‐RCC), eosinophilic variant of clear cell RCC (CCRCC), eosinophilic solid and cystic RCC, eosinophilic vacuolated tumor, fumarate hydratase‐deficient RCC (*FH*‐dRCC), papillary neoplasm with reverse polarity, *TFE3*‐rearranged RCC (*TFE3*‐rRCC), etc. Combined immunostains for CD117, CK7, CK20, AMACR, CA9, FH, GATA3, TFE3, etc. may help to distinguish ONs from these mimics. Most ONs are positive for CD117. Immunoreactivity to CK7 is typically seen in ChRCC, EVT, LGOT, and focally in some oncocytomas and oncocytic variant of PRCCs. Immunoreactivity to CK20 is observed in eosinophilic solid and cystic RCC and may occasionally be seen in oncocytoma. Strong and diffuse immunoreactivity to AMACR is characteristic of oncocytic variant of PRCC. Box‐like CA9 immunoreactivity and CK7 immunonegativity is typical of eosinophilic CCRCC. GATA3 immunoreactivity is seen in papillary neoplasm with reverse polarity and less frequently in ChRCC. Loss of FH expression is diagnostic of *FH*‐dRCC. Strong and diffuse expression of TFE3 supports *TFE3*‐rRCC. In addition, DNA‐ and RNA‐based next‐generation sequencing may further define these differential diagnoses.

Male predominance was observed in renal oncocytoma (M:F = 3:1), whereas other ONs showed no sex‐based differences. There were no statistically significant differences in patient ages, tumor sizes (although malignant RCCs tended to be larger), or prior histories of neoplasms among ON subtypes.

In summary, this study and a previous study^11^ demonstrates that RMB can reliably diagnose and stratify ONs using a combination of cytomorphology from smears or touch preparations, histomorphology from cell blocks or cores, immunohistochemistry, and molecular testing when available. ROM values across cytologic diagnostic categories offer a meaningful framework for guiding management decisions. The high diagnostic performance of RMB, particularly for oncocytoma, supports its clinical utility in avoiding overtreatment and enabling conservative management in appropriate patients. A standardized reporting system for renal cytology will further enhance diagnostic accuracy, facilitate communication between cytopathologist and clinicians, and improve the individualized management of renal oncocytic neoplasms.

## AUTHOR CONTRIBUTIONS

**Xiaoqi Lin:** Conceptualization, methodology, data curation, investigation, validation, formal analysis, project administration, resources, writing—original draft, and writing—review and editing.

## CONFLICT OF INTEREST STATEMENT

The author declares no conflicts of interest.

## Data Availability

The data generated in this study are available from the corresponding author on reasonable request.
